# The chain mediating role of rumination and psychological resilience in symptom burden and kinesiophobia in patients with chronic heart failure

**DOI:** 10.3389/fpsyt.2024.1474715

**Published:** 2024-12-16

**Authors:** Mengqi Li, Lina Cheng, Yu Jiang

**Affiliations:** ^1^ Department of General Medicine/Geriatrics, The Affiliated Central Hospital of Jiangnan University (Wuxi Second People’s Hospital), Wuxi, China; ^2^ College of Wuxi Medical, Jiangnan University, Wuxi, China

**Keywords:** chronic heart failure, kinesiophobia, rumination, resilience, symptom burden, chain mediation

## Abstract

**Objective:**

To explore the chain-mediating role and impact of rumination and psychological resilience on symptom burden and kinesiophobia in patients with chronic heart failure.

**Methods:**

We selected a total of 318 patients with chronic heart failure from a hospital in Wuxi between November 2023 and May 2024 using a convenience sampling method. Various scales and questionnaires assessed general information, symptom burden, resilience, rumination thinking, and kinesiophobia. Statistical analysis was conducted using SPSS and the bootstrap method to examine the chain mediation effect.

**Results:**

The scores for symptom burden, rumination, psychological resilience, and kinesiophobia in patients with chronic heart failure were 0.81± 0.47, 50.63± 9.02, 23.43± 6.26, and 38.91± 8.01, respectively. Mediation analysis showed that symptom burden had a direct positive predictive effect on rumination (β = 0.475, 95% CI: 0.365–0.584), rumination had a direct negative predictive effect on psychological resilience (β = -0.199, 95% CI: -0.306–0.092), and psychological resilience had a direct negative predictive effect on kinesiophobia (β = -0.273, 95% CI: -0.340–0.206). Rumination and psychological resilience played a chain mediation role between symptom burden and kinesiophobia, with a total effect of 0.606, a direct effect of 0.380, and an indirect effect of 0.226. The mediation effect accounted for 37.29% of the total effect.

**Conclusion:**

Kinesiophobia is at a high level in patients with chronic heart failure. Symptom burden can affect patients’ kinesiophobia through the independent or chain mediation effects of rumination and psychological resilience. Healthcare professionals should actively adopt strategies to address symptom burden and rumination while enhancing psychological resilience to reduce patients’ kinesiophobia.

## 1 Introduction

Chronic Heart Failure (CHF) represents the terminal stage of cardiovascular disease and is one of the leading causes of patient mortality (1). During rehabilitation exercises, patients often experience excessive irrational fear, known as kinesiophobia, because of concerns that physical activity might exacerbate their symptoms. Studies have shown that symptom burden, as a primary source of stress for patients with CHF, is a significant positive predictor of kinesophobia (2). The persistent presence of symptom burden may lead to repetitive negative thoughts about emotional distress and stressful events (3), further reinforcing the development of kinesiophobia (4). This, in turn, reduces patients’ confidence and initiative in engaging in rehabilitation exercises. Rumination, defined as a spontaneous and repetitive focus on negative events following stressful experiences, is a maladaptive coping strategy (5). Oncology research has identified rumination as a significant mediator between symptom burden and kinesiophobia (6). Studies on cardiovascular disease have similarly demonstrated that maladaptive coping strategies negatively predict the development of psychological resilience (7, 8), suggesting that rumination may inversely predict resilience. Psychological resilience, the capacity for positive adaptation in adversity (9), is believed to buffer kinesiophobia in patients. Thus, this study hypothesized that the symptom burden experienced by patients with CHF as a chronic stressor may lead to decreased psychological resilience by altering their coping mechanisms. Additionally, resilience was hypothesized to predict kinesiophobia negatively. According to stress-coping theory (10), when faced with significant stressors such as symptom burden, patients adopt different coping strategies, including rumination or resilience. Changes in these strategies and the availability of social resources such as kinesiophobia influence mental health outcomes. Based on this theoretical framework, rumination and psychological resilience are likely key mediators of symptom burden and kinesiophobia.

In summary, this study proposes the following hypothesis: the impact of symptom burden on kinesiophobia in patients with CHF is mediated through a chain mechanism involving rumination and psychological resilience. Existing research on kinesiophobia in patients with CHF has primarily focused on its prevalence and influencing factors (11, 12), with no studies exploring the relationships between symptom burden, rumination, resilience, and kinesiophobia. This study aimed to validate the mediating roles of rumination and resilience between symptom burden and kinesiophobia, thereby providing novel insights for the development of intervention strategies to address kinesiophobia and improve cardiac rehabilitation outcomes.

## 2 Materials and methods

### 2.1 Design and participants

A convenience sampling method used chronic heart failure patients hospitalized at a tertiary hospital in Wuxi between November 2023 and May 2024 as the research subjects. The inclusion criteria were: (1) patients must meet the chronic heart failure diagnostic criteria of the “Chinese Guidelines for the Diagnosis and Treatment of Heart Failure 2024” (13); (2) heart function classification between NYHA II and III; (3) Patients with rehabilitation exercise plans designed by healthcare professionals; (4) age ≥18 years, with basic communication ability, and voluntary participation in the study with informed consent signed. The exclusion criteria were: (1) patients who had undergone heart transplantation or valve replacement surgery; (2) those with a history of severe mental illness, malignant tumors, or severe organ dysfunction; (3) patients with exercise contraindications..

The sample size was determined using G*Power software (version 3.1.9.7) and the linear model calculation method (14). The parameters included a power level of 0.9, an alpha level of 0.05, a moderate effect size of 0.15, and 17 predictor variables (symptom burden, rumination, resilience, demographic, and clinical variables). The sample size required to meet the demands of the multiple regression model was 179. Considering the potential inefficiency rate of 20%, the required number of participants was adjusted to 215. Ultimately, 318 patients with CHF were deemed eligible for inclusion in the study.

### 2.2 Instruments

Demographic Data Questionnaire: This questionnaire was designed based on a comprehensive review of the literature and expert opinions from clinical nursing specialists. It includes questions on patients’ age, gender, education level, heart function classification, clinical staging, and other sociodemographic and clinical disease information.

Memorial Symptom Assessment Scale for Heart Failure (MSAS-HF): This scale evaluates the presence and severity of symptoms experienced by heart failure patients over the past week. It comprises 32 items divided into three subscales: physiological, psychological, and heart failure symptoms (15). The Likert 4-point scale is employed, where higher scores indicate a greater symptom burden. In this study, the scale showed a Cronbach’s α coefficient of 0.855.

Chinese Version of the Tampa Scale for Kinesiophobia (TSK-SV Heart): Adapted by the Swedish scholar Back and translated into Chinese by Lei Mengjie, this scale is designed to evaluate the level of fear related to exercise in cardiac patients. It comprises 17 items across four dimensions (16), with scores exceeding 37 indicating the presence of kinesiophobia and higher scores reflecting greater severity. In this study, the Cronbach’s α coefficient was 0.850.

Ruminative Response Scale (RRS): This scale, translated and revised by Han Xiu and Yang Hongfei, was originally developed by Nolen-Hoeksema (17). It comprises 22 items and uses a Likert 4-point scale to measure three dimensions: symptom rumination, compulsive thinking, and reflective pondering. In this study, the Cronbach’s α coefficient was 0.819.

Connor-Davidson Resilience Scale Short Form (CD-RISC-10): Revised by Campbell-Sills and Stein in 2007 (18), this scale comprises 10 items with a 5-point rating scale from 0 (not at all) to 4 (always), with a total score range of 0 to 40. Higher scores indicate greater psychological resilience. The Chinese version has shown high reliability and validity, with Cronbach’s α coefficients ranging from 0.88 to 0.91. In this study, the Cronbach’s α coefficient was 0.836.

### 2.3 Data collection

Before the survey commenced, all research team members received training and evaluation on topics including the study objectives, use of measurement scales, data collection and organization, and patient communication techniques to ensure standardized procedures. During data collection, patients were provided with a quiet environment, and the purpose of the study, the content of the scales, and the completion guidelines were explained using standardized instructions. For patients with lower educational levels, researchers provided verbal explanations or assistance in completing the scales as needed while avoiding any leading prompts to ensure the authenticity and reliability of the questionnaires. After completing the scales, the researchers collected them on-site and checked for any missing responses or obvious logical errors, promptly verifying these with the respondents. Additionally, a double-entry and double-checking process was implemented to ensure data accuracy.

### 2.4 Data analyses

Data analysis was conducted using SPSS 26.0. Before conducting statistical analyses, we tested the normality of the data using P-P plots and the Kolmogorov-Smirnov test. Categorical data were described using frequencies and percentages, whereas continuous data were presented as means and standard deviations. Descriptive statistics for categorical variables were presented as frequencies and percentages, whereas continuous variables were described using means and standard deviations. Spearman correlation analysis was utilized to explore relationships among rumination, psychological resilience, symptom burden, and kinesophobia in CHF patients. In exploring the differences of these four variables across different demographic characteristics, one-way analysis of variance (ANOVA) or independent samples t-test was used. Significant factors identified from the one-way analysis were integrated as control variables in a chain mediation effect test using the SPSS Process plugin and Bootstrap method. This analysis was to investigate potential mediation effects among the variables. All statistical analyses were carried out at a significance level of α = 0.05, ensuring the precision and dependability of the outcomes.

## 3 Results

### 3.1 Common method bias and collinearity tests

Harman’s single-factor test was used to assess common method bias, revealing 23 common factors with eigenvalues greater than 1. The largest factor explained 20.010% of the variance, which is below the 40% threshold, indicating that common method bias did not severely affect the results. This enhances the reliability and validity of the study’s findings. Collinearity analysis showed tolerance values ranging from 0.506 to 0.581 (< 1) and variance inflation factors (VIF) ranging from 1.720 to 1.976 (<10), indicating that there were no collinearity issues among the independent variables.

### 3.2 Correlation analysis and demographic difference

Pearson correlation analysis was conducted on symptom burden, rumination, psychological resilience, and kinesophobia, as shown in **Table, 1**. The results indicated a significant positive correlation between symptom burden and kinesophobia. Both rumination and symptom burden were significantly positively correlated with kinesophobia, while psychological resilience was significantly negatively correlated with kinesophobia, symptom burden, and rumination. This suggests that patients with higher psychological resilience are better able to manage disease-related stress, reducing both and symptom burden. The relationships among the variables support subsequent hypothesis testing.

**Table, 1:** Descriptive statistical results for each variable and the correlation matrix (r) of variables.

Variable	$\bar{x}$ ±s	1	2	3	4
1 Kinesophobia	38.91 ± 8.01	1			
2 Symptom burden	0.81 ± 0.47	0.796**	1		
3 Ruminative thinking	50.63 ± 9.02	0.712**	0.609**	1	
4 Resilience	23.43 ± 6.26	-0.751**	-0.628**	-0.552**	1

*P<0.05, **P<0.001, the same below

Independent samples t-tests and one-way ANOVA were used to examine the differences in variables based on demographic factors. The results showed significant differences in factors such as age, gender, living arrangements, primary caregiver, family monthly income, education, medical payment method, number of comorbidities, NYHA class, number of hospitalizations, and disease duration (P < 0.05), as shown in **Table, 2**. Therefore, all variables that are statistically significant will be included as control variables in the regression analysis, as shown in **Table, 3**.

**Table, 2:** Differences in variable scores among CHF patients with different demographic characteristics.

Variable	Group	N(%)	Kinesophobia (Mean ± SD)	F/t	P
Age	18-55	31 (9.75)	35.16 ± 7.19	2.950	0.033
	55-64	70 (22.01)	38.84 ± 7.93		
	65-74	102 (32.08)	39.93 ± 8.29		
	≥75	115 (36.16)	39.94 ± 7.81		
Gender	Male	200 (62.89)	37.10 ± 7.31	-5.493	<0.001
	Female	118 (37.71)	41.98 ± 8.23		
Residence	Rural	75 (23.58)	37.40 ± 6.89	2.521	0.058
	Town	84 (26.42)	38.20 ± 8.59		
	County	7 (2.20)	36.41 ± 8.22		
	City	152 (47.80)	40.14 ± 8.07		
Living arrangement	Living with spouse and children	64 (20.13)	41.58 ± 6.29	6.720	0.002
	Living with children	7 (2.20)	37.86 ± 9.39		
	Living with spouse	223 (70.13)	37.68 ± 7.70		
	Living alone	24 (7.55)	43.50 ± 11.16		
Primary caregiver	Spouse	281 (88.36)	38.33 ± 7.98	6.466	0.002
	Children	21 (6.60)	43.62 ± 6.84		
	Others	16 (5.03)	42.81 ± 7.17		
Occupation	Unemployed	2 (0.63)	39.00 ± 12.73	1.585	0.178
	Manual labor	28 (8.81)	37.71 ± 8.21		
	Intellectual labor	13 (4.09)	34.62 ± 9.57		
	Retired	220 (69.18)	39.53 ± 7.96		
	Other	55 (17.30)	38.05 ± 7.42		
Family monthly income, RMB	<1000	17 (5.35)	42.59 ± 8.34	3.466	0.009
	1001-3000	4 (14.47)	41.93 ± 8.34		
	3000-6000	188 (59.12)	38.06 ± 7.83		
	6001-9000	51 (16.04)	38.76 ± 6.87		
	>9000	16 (5.03)	36.75 ± 9.70		
Education	Primary school or below	84 (26.42)	44.71 ± 5.69	30.849	<0.001
	Junior high school	114 (35.85)	39.74 ± 8.08		
	High school/vocational school	84 (26.42)	33.85 ± 6.30		
	College	19 (5.97)	35.00 ± 5.20		
	University or above	203 (63.84)	37.73 ± 7.19		
Medical payment method	Employee health insurance	12 (3.77)	50.67 ± 6.85	15.684	<0.001
	Out-of-pocket and others	64 (20.14)	37.91 ± 8.12		
	Urban resident health insurance	39 (12.26)	43.00 ± 8.24		
	New rural cooperative medical scheme	4 (1.26)	30.50 ± 6.61		
Number of comorbidities	0	28 (8.81)	38.43 ± 7.71	11.770	<0.001
	1	86 (27.04)	35.29 ± 7.94		
	2	200 (62.89)	40.70 ± 7.50		
	≥3	185 (58.18)	37.88 ± 7.80		
NYHA class	II	133 (41.82)	40.35 ± 7.83	-2.740	0.006
	III	15 (4.72)	43.07 ± 6.35		
BMI	<18.5	142 (44.65)	38.43 ± 7.86	2.294	0.103
	18.5-23.9	161 (50.63)	38.94 ± 8.21		
	≥24.0	296 (93.08)	38.07 ± 7.44		
HF hospitalization within the last 1 year, time(s)	1-2	22 (6.92)	50.18 ± 7.01	-7.398	<0.001
	≥3	209 (65.72)	41.47 ± 7.21		
HF duration, years	<5	109 (34.28)	34.00 ± 7.17	8.788	<0.001
	≥5	31 (9.75)	35.16 ± 7.19		

NYHA, New York Heart Association; BMI, Body Mass Index; HF, Heart Failure; SD, standard deviation.

**Table, 3:** Regression analysis of the mediation model of rumination and resilience between symptom burden and kinesiophobia.

Regression Equation		Overall Fit Indices			Regression Coefficient Significance	
Outcome Variable	Predictor Variable	R	R^2^	F	β(95%CI)	t
Rumination	Symptom Burden	0.658	0.433	19.411	0.475(0.365~0.584)	8.516**
Resilience	Symptom Burden	0.699	0.489	22.369	-0.358(-0.474~-0.242)	-6.072**
	Rumination				-0.199(-0.306~-0.092)	-3.650**
Kinesiophobia	Symptom Burden	0.906	0.821	99.190	0.380(0.307~0.453)	10.266**
	Rumination				0.215(0.150~0.280)	6.523**
	Resilience				-0.273(-0.340~-0.206)	-8.037**

**P<0.001.

### 3.3 Chain mediation effect analysis

The PROCESS plugin for SPSS, with Model 6, was used to conduct the chain mediation effect analysis. In this analysis, kinesiophobia was set as the dependent variable, symptom burden as the independent variable, and ruminative thinking and resilience as the mediator variables. Factors with statistical significance from the univariate analysis were included as control variables. Using a non-parametric bootstrap method with 5000 resamples, the mediation effect was tested, and a 95% confidence interval was calculated. The bootstrap results indicated that ruminative thinking and resilience mediate the relationship between symptom burden and kinesiophobia in CHF patients, with a total effect of 0.606 (**Table, 4**). The results show that symptom burden has a direct positive predictive effect on ruminative thinking (β=0.475, P<0.001), ruminative thinking has a direct negative predictive effect on resilience (β=-0.199, P<0.001), and resilience has a direct negative predictive effect on kinesiophobia (β=-0.273, P<0.001) (**Figure, 1**).

**Table, 4:** Mediation effect model of rumination and psychological resilience in the relationship between symptom burden and kinesiophobia in CHF patients.

Mediation Effect Path	Effect Value	Boot SE	Boot LLCI	Boot ULCI
Total Effect	0.606	0.038	0.532	0.680
Direct Effect	0.380	0.037	0.307	0.453
Indirect Effect	0.226	0.029	0.172	0.284
Ind1: Symptom Burden → Ruminative Thinking → Kinesiophobia	0.102	0.020	0.065	0.145
Ind2: Symptom Burden → Resilience → Kinesiophobia	0.098	0.021	0.061	0.142
Ind3: Symptom Burden → Ruminative Thinking → Resilience → Kinesiophobia	0.026	0.008	0.012	0.044

**Figure, 1:**
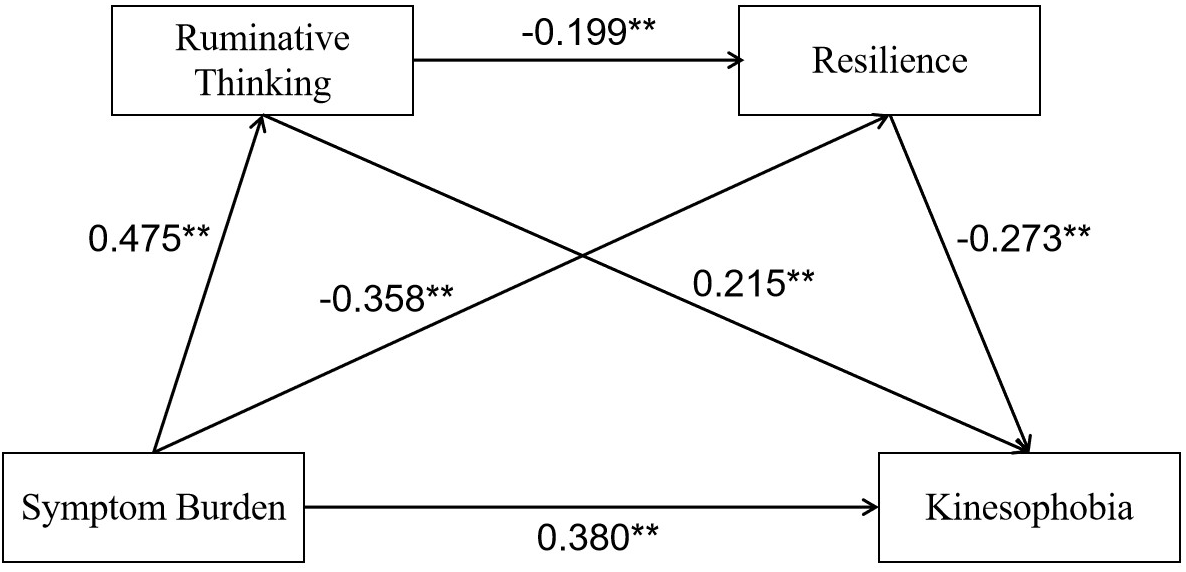
The mediation model diagram of symptom burden and exercise fear.

## 4 Discussion

Heart failure, identified as a chronic progressive cardiovascular condition, presents a significant burden on individuals and their families. Regular aerobic exercise is crucial for the cardiac rehabilitation of patients with chronic heart failure. However, in practice, the actual participation rate in exercise rehabilitation among heart failure patients is not ideal (19). Studies have confirmed that kinesiophobia is one of the main obstacles preventing patients from engaging in physical activities (20) and participating in cardiac rehabilitation, with an incidence rate of 20% to 76% (21, 22). Furthermore, negative emotions increase the risk of cardiovascular damage by 2 to 2.5 times (23). A total of 318 patients with rehabilitation exercise plans developed by healthcare professionals were screened, among whom 193 were identified as having kinesiophobia, with a representativeness rate of 60.69%. Therefore, reducing patients’ symptom burden, decreasing rumination thinking, and enhancing psychological resilience are essential for alleviating kinesiophobia and lowering the risk of recurrence in heart failure patients.

### 4.1 Analysis of symptom burden, rumination thinking, psychological resilience, and kinesiophobia in CHF patients

The results of this study indicate that symptom burden is significantly associated with exercise phobia, and symptom burden, as an initial stressor, positively predicts exercise phobia. Severe symptom burden suggests that patients experience more physical pain and treatment side effects caused by the disease. Pain-related fear has been identified as a predictor of disability in patients, and it is considered more predictive than pain intensity or structural damage (24). According to the “fear-avoidance” model, when pain events are perceived as threatening, catastrophic thoughts arise, and patients may believe that exercise and physical activity will lead to further pain and injury, thereby exacerbating exercise phobia.

In this study, the kinesiophobia score was (38.91 ± 8.01), similar to the results of Qin Jingwen et, al. (25) in their research on elderly patients with chronic heart failure, indicating a high level of kinesiophobia. This confirms that kinesiophobia as a common psychological issue among CHF patients, severely affecting their cardiac rehabilitation process. The direct impact of symptom burden on kinesiophobia behavior in CHF patients accounted for 62.71% of the total effect, with a disease burden score of (0.81 ± 0.47), which was lower than the results of Li et, al. (26) in their study on symptom burden in heart failure patients. This difference may be due to variations in the types of subjects surveyed. This study included hospitalized patients with chronic heart failure at heart function levels II-III, whose symptoms were relatively stable, resulting in a lower overall symptom burden score. Furthermore, the total rumination thinking score of CHF patients in this study was (50.63 ± 9.02), with the symptom rum ination dimension score at (27.62 ± 5.73), the compulsive thinking dimension score at (10.40 ± 2.33), and the reflection dimension score at (12.61 ± 2.62), all higher than the national norms (17). This indicates that most CHF patients are prone to negative cognitive patterns and have relatively severe rumination thinking. This is due to the current situation of CHF patients, who are primarily receiving supportive treatment and lacking specific therapies. Additionally, long-term treatment and recurrent conditions lead patients into deep rumination thinking. Watkins et, al. (27) pointed out that rumination not only exacerbates patients’ anxiety and depression but also weakens their ability to handle stress and solve problems, hindering effective stress adaptation and rehabilitation processes. The psychological resilience score in this study was (23.43 ± 6.26), lower compared to the scores reported in the study by Ma et, al. (28). This difference may be because CHF patients face long-term treatment challenges and condition fluctuations, which continuously deplete their psychological resources, thereby weakening their psychological resilience.

### 4.2 The independent mediating effects of rumination and psychological resilience on the relationship between symptom burden and exercise phobia in CHF patients

This study found that rumination has a significant mediating effect between symptom burden and exercise phobia. Mechanistically, rumination leads patients to repeatedly focus on and amplify their symptom burden and disease risks, resulting in excessive worry about the consequences of exercise (29). Patients may excessively fear that exercise could worsen their condition or cause discomfort, thus developing a strong avoidance attitude toward physical activity. Research has shown that symptom burden influences negative cognitive perceptions of exercise by increasing rumination (30), and this amplification effect significantly elevates patients’ exercise phobia. Furthermore, rumination not only directly exacerbates exercise phobia but may also indirectly strengthen it by worsening patients’ emotional states (31), such as anxiety and depression. This suggests that rumination is a central driving force in a negative cycle that deeply impacts both the cognitive and emotional aspects of exercise phobia.

The results of this study also indicate that psychological resilience has a significant mediating effect between symptom burden and exercise phobia. Mechanistically, psychological resilience can reduce the negative impact of symptom burden on exercise phobia by enhancing individuals’ coping abilities and self-efficacy (32). When psychological resilience is high, patients are better able to confront the physical discomfort and psychological stress caused by heart failure (33), reducing excessive concern about exercise-related risks. Additionally, patients with higher psychological resilience exhibit stronger regulatory abilities (34) when faced with rumination, further reducing their fear of exercise. Other studies have also demonstrated that the mediating effect of psychological resilience not only directly lowers exercise phobia levels but also indirectly alleviates it by modulating other negative psychological factors, such as anxiety and depression (35). Therefore, in intervention practices, improving rumination and enhancing psychological resilience should be core strategies to mitigate exercise phobia.

### 4.3 Mediating effects of ruminative thinking and resilience

The mediation analysis results indicate that rumination thinking and psychological resilience exhibit a chain mediation effect between symptom burden and kinesiophobia in CHF patients (effect value = 0.026), with the indirect effect accounting for 34.07% of the total effect. As CHF patients experience increased symptom burden due to declining heart function, negative symptom experiences intensify their negative emotions and insufficient understanding of the disease, causing patients to focus more on the potential negative consequences of exercise. This leads to a continuous review and amplification of past painful experiences, resulting in rumination thinking. Additionally, the inherent characteristics of chronic heart failure, such as its irreversibility and difficulty to cure (36), exacerbate the negative emotional experiences of CHF patients, adversely affecting their mental health and leading to a decline in psychological resilience. This dual predicament—physical impairment and psychological deterioration—jointly contributes to patients’ kinesiophobia. The results of this study demonstrated that rumination and psychological resilience serve as a chain mediation between symptom burden and kinesophobia in CHF patients. The simple mediation effect value for rumination was 0.102, while that for psychological resilience was 0.098.

As the condition stabilizes, heart failure patients become the primary executors of disease management, requiring active or passive self-regulation to readjust to life after illness. During this process, kinesiophobia often emerges as a significant obstacle. Currently, common interventions for kinesiophobia, such as graded exposure therapy (37), social support (38), cognitive behavioral therapy (39), and internet-based remote rehabilitation therapy (40), each have unique advantages and applicable scopes. However, stepped care model, due to its gradual progression, accessibility, and low cost, may have more significant effects in helping patients gradually overcome kinesiophobia and improve psychological resilience (41). This method not only focuses on the physiological recovery of patients but also emphasizes the adjustment and enhancement of their psychological state. By progressively increasing the intensity of the interventions, patients gradually regain confidence and ability in physical activity through active participation. This approach not only helps patients overcome kinesiophobia but also enhances their disease management skills (42), enabling them to better cope with various challenges and stressors in daily life. This positive psychological state plays a crucial role in promoting long-term recovery and improving patients’ quality of life.

## 5 Limitations

This study has several limitations that should be acknowledged. First, the cross-sectional design of the study limited the ability to establish causal relationships between variables. Future research should employ longitudinal designs to validate these findings further. Additionally, traumatic events and the presence of anxiety or depression may have a negative impact on kinesiophobia; however, these factors were not measured in this study, warranting further investigation. Finally, due to time and resource constraints, participants were recruited from a single hospital within the city, which may limit the generalizability of the findings. Future studies should adopt a multicenter design and include a larger sample size to enhance the generalizability and external validity of the results.

## 6 Conclusion

The results of this study indicated that kinesiophobia is relatively high among patients with CHF. Symptom burden, rumination, and psychological resilience had both direct and indirect effects on kinesiophobia, aligning with the stress-coping theoretical framework. Moreover, rumination and psychological resilience served as sequential mediators in the relationship between symptom burden and kinesiophobia in patients with CHF. The clinical significance of this study lies in its in-depth investigation of the mechanisms influencing kinesiophobia in patients with CHF, which provides novel insights for developing intervention strategies to alleviate kinesiophobia and improve cardiac rehabilitation management outcomes.

## Data Availability

The raw data supporting the conclusions of this article will be made available by the authors, without undue reservation.
